# Dual In Vivo and In Silico Exploration of Jet A‑1 Fuel Toxicity

**DOI:** 10.1021/acsomega.5c10984

**Published:** 2026-09-25

**Authors:** Yuli Subiakto, Yahdiana Harahap, Widya Hardiyanti, Budi Sumaryono, Habibie Habibie, Muh. Arfandy Gunawan, Tesia Aisyah Rahmania, Muhammad Raihan, Nadila Pratiwi Latada, Firzan Nainu

**Affiliations:** † Department of Military Pharmacy, Faculty of Military Pharmacy, Republic of Indonesia Defense University, Bogor 16815, Indonesia; ‡ Unhas Fly Research Group, Faculty of Pharmacy, Hasanuddin University, Makassar 90245, Indonesia; § Study Program of Pharmacy, Faculty of Medicine and Health Sciences, Universitas Muhammadiyah Makassar, Makassar 90221, Indonesia; ∥ Department of Pharmacy, Faculty of Pharmacy, 64739 Hasanuddin University, Makassar 90245, Indonesia; ⊥ Chemical Biology and Medicinal Chemistry Research Group, Faculty of Pharmacy, 355648 Hasanuddin University, Makassar 90245, Indonesia; # Department of Pharmaceutical Science and Technology, Faculty of Pharmacy, Hasanuddin University, Makassar 90245, Indonesia

## Abstract

Occupational and environmental exposure to aviation fuel requires rigorous toxicity evaluation to protect public health. This study assessed the neurotoxic potential of Jet A-1 aviation fuel using a combined in vivo and in silico approach. In *Drosophila melanogaster*, continuous exposure to Jet A-1 significantly reduced survival and locomotor activity in both sexes. RT-qPCR analysis revealed increased expression of *AChE*, *rpr*, *sod1*, *sod2*, and *cat*, indicating cholinergic dysfunction, activation of the apoptotic pathway, and increased oxidative stress. Given the high naphthalene content in Jet A-1 fuel, network pharmacology analysis identified approximately 129 complementary targets associated with naphthalene derivative-induced neurotoxicity. These targets were enriched in pathways related to apoptosis, oxidative stress, impaired proteostasis, and inflammatory signaling, suggesting a multifactorial neurotoxic response. Further molecular docking analysis demonstrated strong interactions between Jet-A1 and key proteins such as AKT1, EGFR, PTEN, CSK, SHG, and Decay, corroborating the in vivo findings. Together, these results elucidate the mechanistic basis of Jet A-1-induced neurotoxicity and reinforce the value of *D. melanogaster* as a reliable in vivo model for high-throughput toxicological studies of chemical exposure.

## Graphical abstract

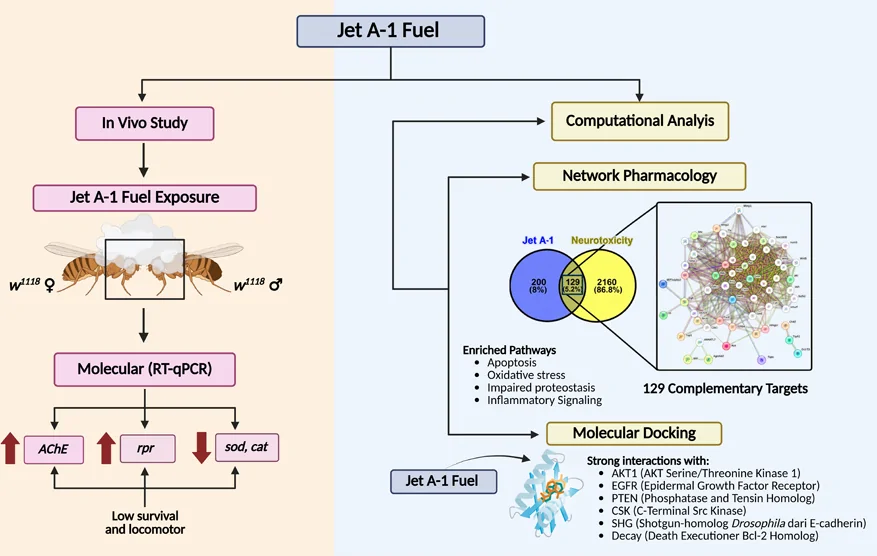

## 1 Introduction

Airports are among the major sources of pollution in the aviation industry. One of the significant health risks comes from aircraft refueling activities, which pose serious exposure hazards to technical personnel.^1^ Aircraft fuel emissions contain volatile organic compounds (VOCs) and fine particles composed of inorganic carbon cores, polycyclic aromatic hydrocarbons (PAHs), and heavy metals. These compounds have the potential to cause various health issues, including skin irritation, cardiovascular disorders, immune dysfunction, and cancer.^2,3^ Jet A-1 is the most commonly used type of aviation fuel worldwide. It is a complex mixture of hydrocarbons, including aromatic compounds such as benzene, toluene, and xylene, as well as chemical additives designed to enhance performance and thermal stability. These chemical components are potentially toxic when organisms or the environment exposed to significant concentrations. However, research on the toxicity of Jet A-1 fuel, particularly its effects on biological systems, remains limited.^4^

In preclinical studies, toxicity testing of chemical compounds typically involves the use of animal models. Traditional models such as mice or rats, while biologically relevant, are time-consuming and costly especially for inhalation-based toxicity studies. Therefore, alternative animal models that are more efficient yet still biologically meaningful are needed. *Drosophila melanogaster*, or the fruit fly, has been widely used in toxicological research due to its short life cycle, low maintenance cost, high reproductive rate, and genetic similarity to humans (approximately 75%).^5,6^ These features make *D. melanogaster* an ideal model for initial chemical toxicity screenings, including for compounds such as Jet A-1 fuel.

In addition to animal-based testing, computational methods have emerged as valuable tools in toxicology and drug discovery. Techniques such as network pharmacology can be employed to identify key molecular targets and biological pathways associated with toxic compounds,^7,8^ while molecular docking enables the prediction of binding interactions between toxicants and specific proteins at the molecular level.^9^ Applying these approaches to Jet A-1 components, such as naphthalene, may provide mechanistic insights into their potential neurotoxic or systemic toxic effects. When combined with in vivo models like *D. melanogaster*, these computational methods strengthen the predictive power of toxicity studies and accelerate the identification of health risks related to Jet-A1 fuel exposure.

Toxicity testing of Jet A-1 fuel is therefore critical to support the development of health and safety regulations, especially for refueling and aircraft maintenance personnel. Considering the potential health hazards of Jet A-1 and the importance of protecting airport workers, this study aims to evaluate the effects of Jet A-1 exposure on *D. melanogaster* as an initial screening model for chemical toxicity, complemented by computational analyses using network pharmacology and molecular docking. The findings are expected to provide preliminary data for understanding the health risks of jet fuel exposure and to contribute to the development of safer aviation fuels in the future.

## 2 Materials and Methods

### 2.1 Materials

This study utilized aviation Fuel (Jet A-1) (CAS: 64742-81-0) obtained from PT. Pertamina Patra Niaga, Indonesia; the Luna Universal One-Step RT-qPCR Kit (New England Biolabs, Cat. No. E3005S); the Monarch Total RNA MiniprepKit (New England Biolabs, Cat. No. T2110S); and a Nitroblue Tetrazolium Chloride (NBT) reagent kit (CAS: 298-83-9) purchased from Himedia.

### 2.2 In Vivo *Drosophila* Assay

#### 2.2.1 *Drosophila* Stock and Maintenance

*Drosophila melanogaster* *w* ^*1118*^ strain (obtained from the Laboratory of Host Defense and Responses, Kanazawa University, Japan) was used in this study. Flies were maintained under controlled environmental conditions (25 ± 1 °C, 60 ± 5% relative humidity) on a standard cornmeal-based fly food, with a 12 h:12 h light–dark cycle.

#### 2.2.2 Survival Assay of Jet A-1 Fuel Exposure on *Drosophila*

Three-days-old male and female *D. melanogaster* were collected separately and transferred into vials. For the Jet A-1 fuel exposure, 10 flies were placed into a vial and covered with a stopper plugs. Subsequently, 500 μL of Jet A-1 fuel was applied to the top of the plug, which was then sealed with an additional plug. The flies were observed until all individuals had died, with the time of death recorded at 1 min intervals. Each treatment was performed in three replicates, compared to the untreated control (Figure 1).^10^

**Figure 1:**
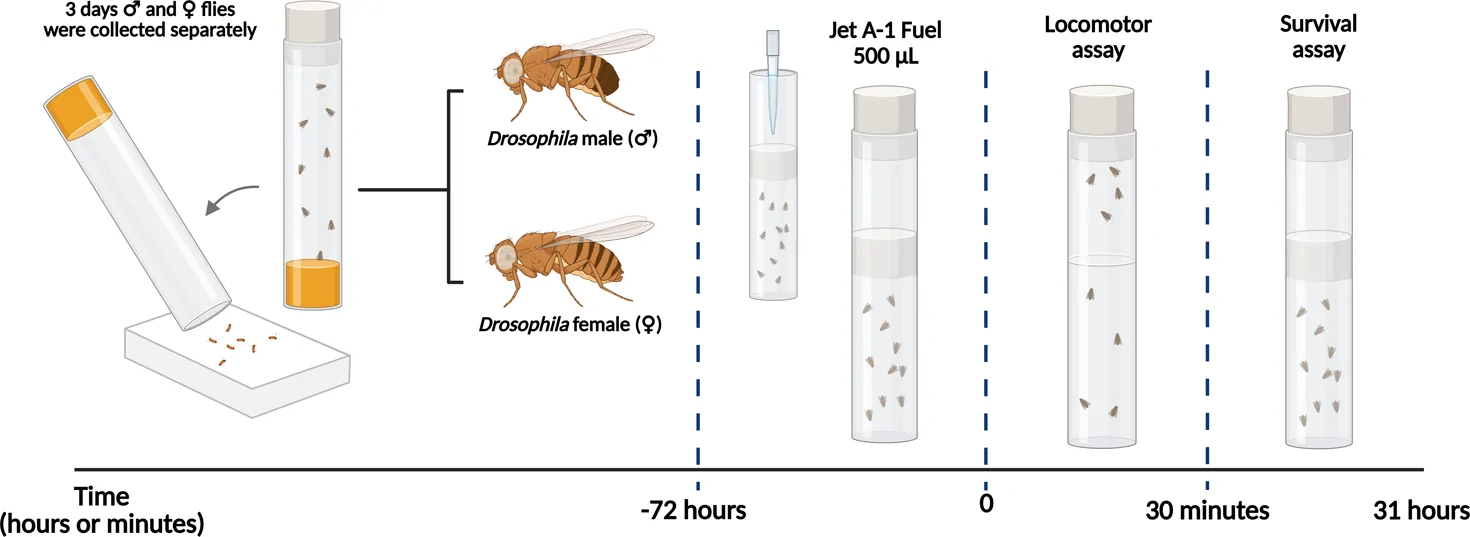
Experimental methods used in this study. Created in BioRender. https://BioRender.com/7y98ssx.

#### 2.2.3 Locomotor Assay

The locomotor ability of *D. melanogaster* was assessed using the negative geotaxis assay. Both male and female flies were separated and each group was exposed to Jet A-1 fuel every 10 min prior to the locomotor test procedur. The flies were then placed into vials measuring approximately 10 cm in height and 2.5 cm in diameter, with 10 flies per group. After a 10 min adaptation period at room conditions, the flies were gently tapped to the bottom of the vial. The time required for the flies to climb was recorded. The assay was performed in three replicates with a 1 min interval between trials. The parameters observed included the percentage of flies able to reach the 6 cm mark within 10 s and the average vertical climbing speed (cm/s). The data obtained were analyzed to compare differences in locomotor performance between the treatment and control groups.

#### 2.2.4 Nitroblue Tetrazolium (NBT) Reduction Assay

Reactive oxygen species (ROS) levels in *Drosophila melanogaster* were quantified using the nitroblue tetrazolium (NBT) reduction assay. This assay is based on the reduction of nitroblue tetrazolium, a yellow dye, by superoxide anions to form water-insoluble blue formazan precipitates, the intensity of which is proportional to ROS levels and can be quantified by measuring absorbance at 595 nm. Briefly, 100 adult flies were collected, and 100 μL of phosphate-buffered saline (PBS) was added. Hemolymph extraction was performed on ice to prevent melanization. A total reaction volume of 300 μL was prepared by adding 200 μL of NBT reagent to the hemolymph sample. The mixture was incubated at room temperature in the dark for 1 h. The reaction was terminated by adding 300 μL of 100% glacial acetic acid. Subsequently, the samples were centrifuged at maximum speed for 1 min, and the absorbance of the supernatant was measured at 595 nm using a spectrophotometer.^11^

#### 2.2.5 Gene Expression Analysis

Five adult *D. melanogaster* *w* ^*1118*^ flies with or without Jet A-1 fuel exposure were placed in treff tubes for total RNA isolation using the Monarch Total RNA Miniprep Kit (New England Biolabs, Inc., U.S.), following the manufacturer’s protocol. Gene expression in *w* ^*1118*^ flies was assessed with RT-qPCR methods using Luna Universal One-Step RT-qPCR Kit (New England Biolabs, Inc., U.S.). The RT-qPCR reactions were conducted in a 10 μL final volume. The cycling conditions included reverse transcription at 50 °C for 10 min, followed by an initial enzyme activation step at 95 °C for 2 min. Amplification proceeded for 40 cycles, with denaturation at 95 °C for 10 s, annealing at 60 °C for 30 s, and extension at 72 °C for 30 s. To confirm the specificity of the amplified products, a melt curve analysis was conducted over a temperature range of 60–95 °C. The primer sequences used in this assay are provided in Table 1. Additionally, the RNA levels of the target gene were assessed using a set of *rp49* primers, which served as an internal reference to normalize the expression levels

**Table 1:** Primers Used in the RT-qPCR Assay

Genes	Forward primer (5′ → 3′)	Reverse primer (5 ′→ 3′)
*sod1*	AGGTCAACATCACCGACTCC	GTTGACTTGCTCAGCTCGTG
*sod2*	TGGCCACATCAACCACAC	TTCCACTGCGACTCGATG
*cat*	TTCCTGGATGAGATGTCGCACT	TTCTGGGTGTGAATGAAGCTGG
*rpr*	ACTGGATCCCAATGGCAGTGGCATTCT	AAAGGATCCTCATTGCGATGGCTTGC
*AChE*	GCCGTGGGCAATGTAATAGT	CGACTCTCCGAACAGTGTCA
*rp49*	GACGCTTCAAGGGACAGTATCTG	AAACGCGGTTCTGCATGAG

### 2.3 Preparation and Conditions Using Gas Chromatograph–Mass Spectrometry Measurements

Jet A-1 fuel of 0.1 g of was added with 5 mL of methanol and then covered with aluminum foil. The mixture was extracted using a sonicator at 70 °C for 20 min, then filtered using a syringe filter and put into a vial. Chromatography tests used a Shimadzu Ultra QP 2010 GC-MS instrument. The instrument conditions were operated at an injector temperature of 250 °C with splitless mode, a pressure of 76.9 kPa and a flow rate of 1.4 mL/min and a ratio of 1:10. The ion source and interface temperatures were 200 and 280 °C, a solvent cut time of 3 min, 400–700 *m*/*z*. The column type was SH-Rxi-5Sil MS, a column length of 30 m with an inner diameter of 0.25 mm. The initial column temperature was 700 °C with a holding time of 2 min and the temperature was increased to 200 °C at a rate of 100 °C/min and the final temperature was 280 °C with a holding time of 9 min at a rate of 50 °C/min so that the total analysis time was 30 min. The obtained chromatogram data were interpreted using the NIST 20 library on the instrument application.

### 2.4 Network Pharmacology Analysis

#### 2.4.1 Identification of Gene Target

Major compounds of Jet A-1 fuel were used for analysis. Information about the targets associated with the major compound of Jet A-1 fuel was retrieved from the CTD database (http://ctdbase.org/). We collected all the targets, deleted duplicates and got candidate toxic compounds of Jet A-1 Fuel and potential target genes. We identified potential neurotoxicity targets by searching the GeneCards database (https://www.genecards.org)^12^using the keywords “toxic encephalopathy” and “nerve toxicity,” applying a filter of relevance score ≥ 10. The results from both searches were then merged, and duplicate targets were removed to obtain the final list of potential neurotoxicity targets.^13^

#### 2.4.2 Protein–Protein Interaction (PPI)

To identify intersection targets between candidate toxic compound targets and those associated with neurotoxicity, we utilized the Venny 2.1.0 tool (https://bioinfogp.cnb.csic.es/tools/venny/).^14^ Genes present in both data sets were considered as potential mediators of the neurotoxic effects of Jet A-1 Fuel on neural tissues. All intersecting targets were subsequently uploaded to the STRING database (https://cn.string-db.org/) for protein–protein interaction (PPI) network construction.^15^ Visualization and further analysis of the PPI network were performed using Cytoscape software (version 3.9.1).^16^ Topological parameters for each target within the network were evaluated using the CytoHubba plug-in, with node degree values highlighting the importance of core targets.^17^ Finally, we imported the candidate toxic compounds from Jet A-1 Fuel together with their six core targets into Cytoscape 3.9.1 to generate a compound-target relationship network.^18^ In addition to the Degree value ranking, gene selection was also considered based on their roles in regulating apoptosis mechanisms and involvement in biological responses to stress.

#### 2.4.3 Gene Enrichment Analysis

To explore the potential biological significance of the identified target genes, Gene Ontology (GO) enrichment and Kyoto Encyclopedia of Genes and Genomes (KEGG) pathway analyses were conducted using the STRING database (http://cn.string-db.org).^19^ The input consisted of a list of gene symbols associated with the predicted or known protein–protein interaction (PPI) network.^20^ GO enrichment analysis included the Biological Process (BP), Molecular Function (MF), and Cellular Component (CC) categories,^21^ whereas KEGG pathway enrichment was used to identify relevant signaling and metabolic pathways.^22^ Visual representations of the GO enrichment and KEGG pathway annotations were generated using the SRplot web server.^23^

### 2.5 In Silico Molecular Docking

The molecular structure of compounds Jet A-1 Fuel was retrieved from the PubChem database (https://pubchem.ncbi.nlm.nih.gov/). The canonical SMILES format was converted into a three-dimensional structure file (PDB format) using UCSF Chimera 1.18.3 (University of California, San Francisco, USA).^24^ Ligand preparation was performed by adding polar hydrogen atoms and assigning Gasteiger charges, followed by energy minimization using the “Minimize Structure” tool in UCSF Chimera to optimize geometry and ensure conformational stability prior to docking.^25^ The crystal structures of the target proteins AKT1, EGFR, PTEN, CSK, shg (DE-cadherin), Debcl, bnl, and Decay were obtained from the Research Collaboratory for Structural Bioinformatics Protein Data Bank (http://www.rcsb.org/). Protein preparation was conducted in UCSF Chimera using the “Dock Prep” tool, which included hydrogen addition, correction of missing side chains, charge assignment, and removal of water molecules and other nonessential heteroatoms. The optimized protein structures were then saved in PDB format for subsequent docking analysis.^26^ Molecular docking simulations were carried out using UCSF Chimera 1.18.3 integrated with AutoDock Vina. The binding site was defined by setting the grid box coordinates and dimensions based on the active site of each target protein. The docking protocol generated multiple binding conformations, from which the pose with the lowest binding free energy was selected as the most favorable. The resulting protein–ligand complexes were further analyzed and visualized using Biovia Discovery Studio Visualizer 2024 (Dassault Systèmes, Waltham, MA, USA) to evaluate key molecular interactions.^27^

### 2.6 Data Processing and Statistical Analysis

All data were analyzed using GraphPad Prism 9, based on a minimum of three independent biological replicates. Survival curves were evaluated using the log-rank test and visualized as Kaplan–Meier plots. Locomotor activity data were presented as bar graphs and analyzed using two-way ANOVA. ROS and gene expression levels were displayed as bar graphs and statistically assessed using unpaired *t* tests.

## 3 Results and Discussion

### 3.1 Continuous Exposure to Jet A-1 Fuel Reduces Survival in Both Male and Female Flies

Aviation fuels, including Jet A-1, are complex mixtures of hydrocarbons that may exert toxic effects on living organisms. To assess the acute toxicity of Jet A-1, a survival assay was performed using male and female flies as experimental models. The results showed that exposure to Jet A-1 markedly reduced survival in both male (Figure 2A) and female flies (Figure 2B). All exposed flies died within less than 100 min, whereas the control groups survived for more than 1000 min, displaying a gradual decline in survival. This result indicates that Jet A-1 fuel exerts acute toxicity on flies, with no pronounced differences in mortality rate between both sexes.

**Figure 2:**
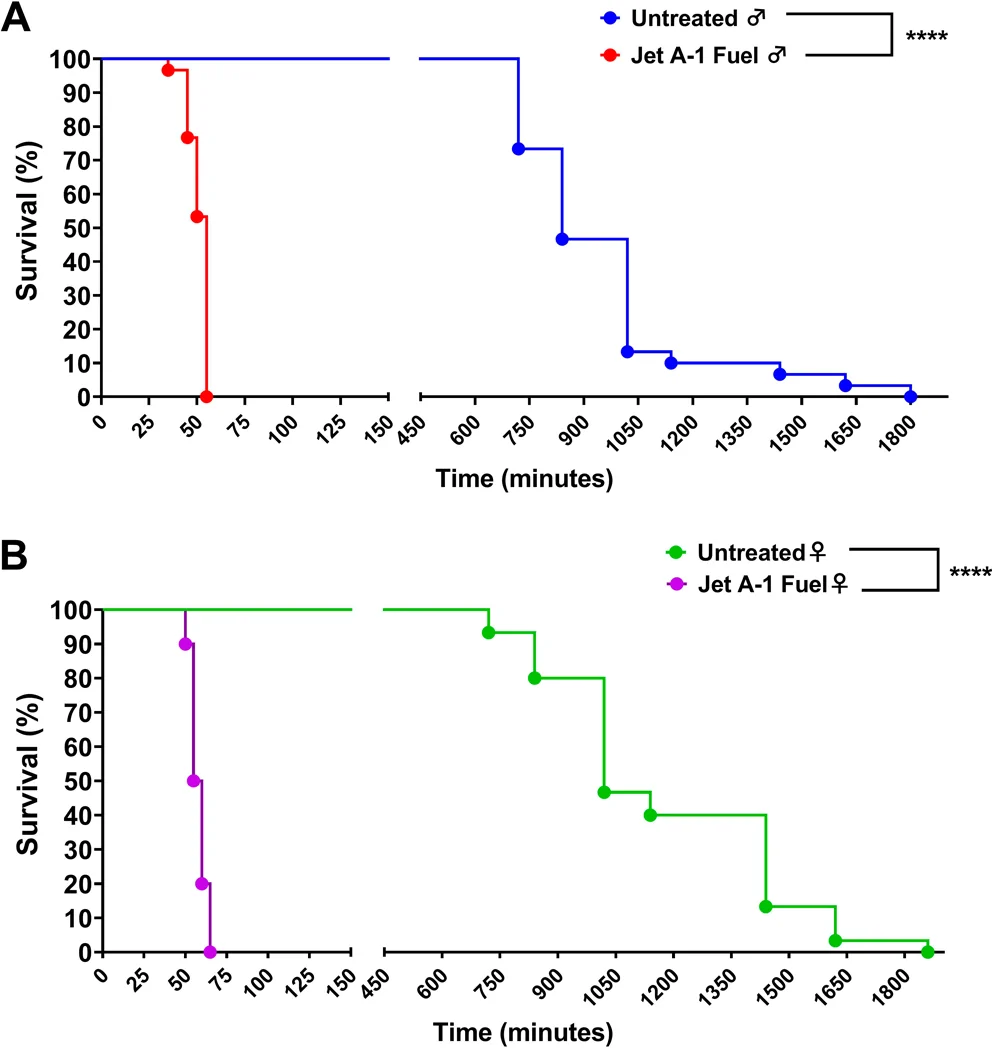
Continuous exposure to Jet A-1 fuel significantly reduced the survival of male (A) and female (B) *Drosophila*. The observed acute toxicity was not sex-dependent, indicating comparable susceptibility between males and females. Statistical significance is indicated as ****p < 0.0001.

To further determine whether the observed toxicity was attributable to naphthalene, one of the aromatic hydrocarbons detected in Jet A-1 fuel, additional survival assays were conducted using naphthalene-only exposure together with the corresponding solvent controls (Supporting Information, Figures S1 and S2). Exposure to naphthalene significantly reduced survival in both male and female flies compared with untreated controls. However, mortality occurred more gradually than in the Jet A-1-treated groups. Similarly, exposure to the solvent controls (acetonitrile-ethanol and DMSO) also resulted in reduced survival relative to untreated flies, although the toxic effects remained less pronounced than those observed following Jet A-1 exposure.

Notably, Jet A-1 exposure produced the most rapid decline in survival, resulting in complete mortality within a shorter period than either naphthalene or solvent treatments. These findings indicate that naphthalene contributes to the overall toxicity of Jet A-1 but cannot fully account for its acute lethal effects. Given that Jet A-1 is a complex mixture of aliphatic and aromatic hydrocarbons, the enhanced toxicity is likely attributable to the combined or synergistic actions of multiple fuel-derived constituents. Such compounds have been reported to induce oxidative stress, mitochondrial dysfunction, membrane damage, and neurotoxicity, ultimately accelerating mortality. Therefore, the severe reduction in survival observed following Jet A-1 exposure likely reflects the cumulative effects of multiple toxic components rather than naphthalene alone.

### 3.2 Jet A-1 Fuel Exposure Induces Neurotoxicity in *Drosophila*

In addition to its detrimental impact on survival rates, the toxicity of Jet A-1 fuel exposure in *Drosophila melanogaster* was further evaluated through locomotor activity assessment. Although the initial response phase (0–10 min) showed no significant alterations, prolonged exposure revealed marked neurotoxic effects, as indicated by a significant decline in fly movement (Figure 3A–B). These reductions in locomotor performance align with previous reports demonstrating that exposure to volatile organic compounds (VOCs) and hydrocarbons present in jet fuel can trigger oxidative stress, impair synaptic transmission, and disrupt nervous system function.^28,29^ Interestingly, a sex-dependent variation was observed, where female flies exhibited delayed sensitivity, with significant locomotor impairments becoming evident only after 20 min of exposure. This finding suggests potential sex-specific differences in xenobiotic metabolism and detoxification enzyme activity that may influence locomotor responses.^30^

**Figure 3:**
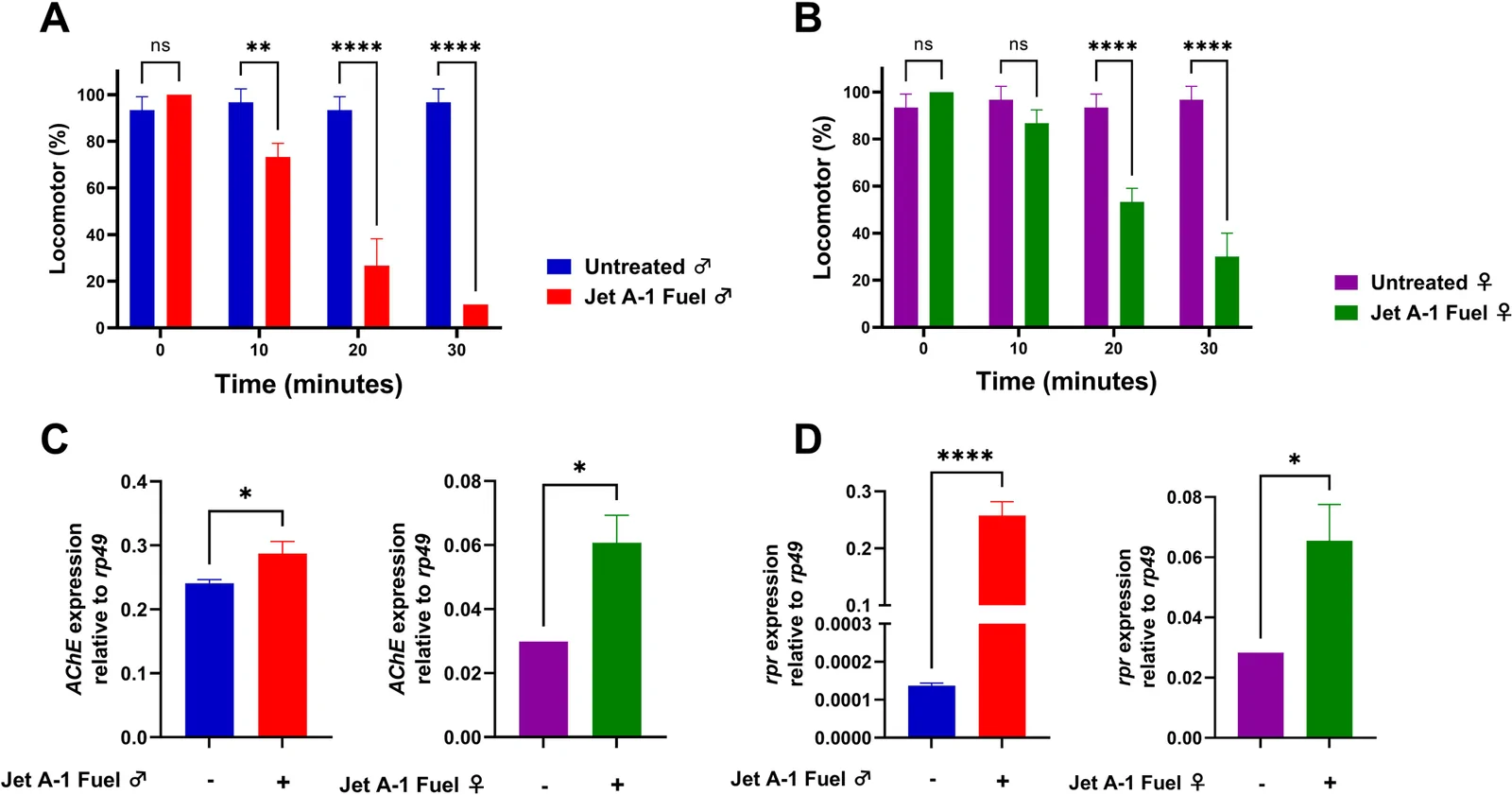
Jet A-1 fuel exposure significantly decreased locomotor activity in both male (A) and female (B) *D. melanogaster* at 20 and 30 min compared with controls. Neurotoxic effects were further supported by the upregulation of *AChE* (C) and *rpr* (D) gene expression. Data are shown as mean ± SEM. Statistical significance is denoted as ns (not significant), *p < 0.05, **p < 0.01, ***p < 0.001, and ****p < 0.0001.

*D. melanogaster* possesses a well-characterized nervous system, genetic tractability, and conserved molecular pathways relevant to human neurobiology.^31^ To further elucidate the neurotoxic effects of Jet A-1 fuel, we examined the expression of the *AChE* gene, which encodes acetylcholinesterase, an enzyme responsible for hydrolyzing acetylcholine within the synaptic cleft. Molecular analysis revealed a significant upregulation of *AChE* expression in both male and female flies following Jet A-1 exposure (Figure 3C). This elevated expression may indicate a reduced level of acetylcholine, which has associated with impaired neuronal homeostasis.^32,33^ Comparable alterations in cholinergic signaling have been documented in insect neurotoxicology models exposed to organophosphate pesticides and aromatic hydrocarbons, highlighting a conserved mechanism of neurochemical disruption.^34^

Additionally, the observed results are supported by the increased expression of *rpr*, encodes Reaper, one of the key pro-apoptotic genes in *D. melanogaster* (Figure 3D). Reaper mediates apoptosis by inhibiting IAPs (Inhibitor of Apoptosis Proteins), thereby facilitating caspase activation and execution of programmed cell death.^35,36^ Activation of this apoptotic cascade typically occurs in response to oxidative stress, cellular damage, or mitochondrial dysfunction induced by xenobiotic exposure. The enhanced apoptotic response at the molecular level thus might reinforces the potential neurotoxic effects of the Jet A-1 fuel.^37^

### 3.3 Jet A-1 Fuel Exposure Increases ROS and Affects the Expression of Endogenous Antioxidant-Related Genes in Male and Female Flies

We further investigated the effect of Jet A-1 exposure on cellular oxidative stress by measuring reactive oxygen species (ROS) in both male and female flies, using NBT assay. The results revealed a significant increase in the formation of water-insoluble blue formazan precipitates, quantified by measuring absorbance at 595 nm, indicating elevated ROS levels following Jet A-1 exposure (Figure 4A). This increase is consistent with the potential toxicity of hydrocarbon mixtures containing aromatic compounds, which may promote oxidative stress through enhanced ROS production.^38,39^ Under normal physiological conditions, the upregulation of ROS levels is counter balanced by endogenous antioxidant activity that maintain cellular redox homeostasis.^40^ However, molecular analysis revealed a suppression of endogenous antioxidant activity following Jet A-1 exposure, indicating a disruption in the antioxidant defense genes expression such us *sod1*, *sod2* and *cat* (Figure 4B–D). This reduction may result in excessive ROS accumulation, leading to cellular damage and the activation of apoptotic pathways.^38^ Collectively, these findings validate the neurotoxic effects of Jet A-1 fuel exposure, primarily mediated through oxidative stress induction and the disruption of antioxidant gene regulation in *D. melanogaster* of both sexes.

**Figure 4:**
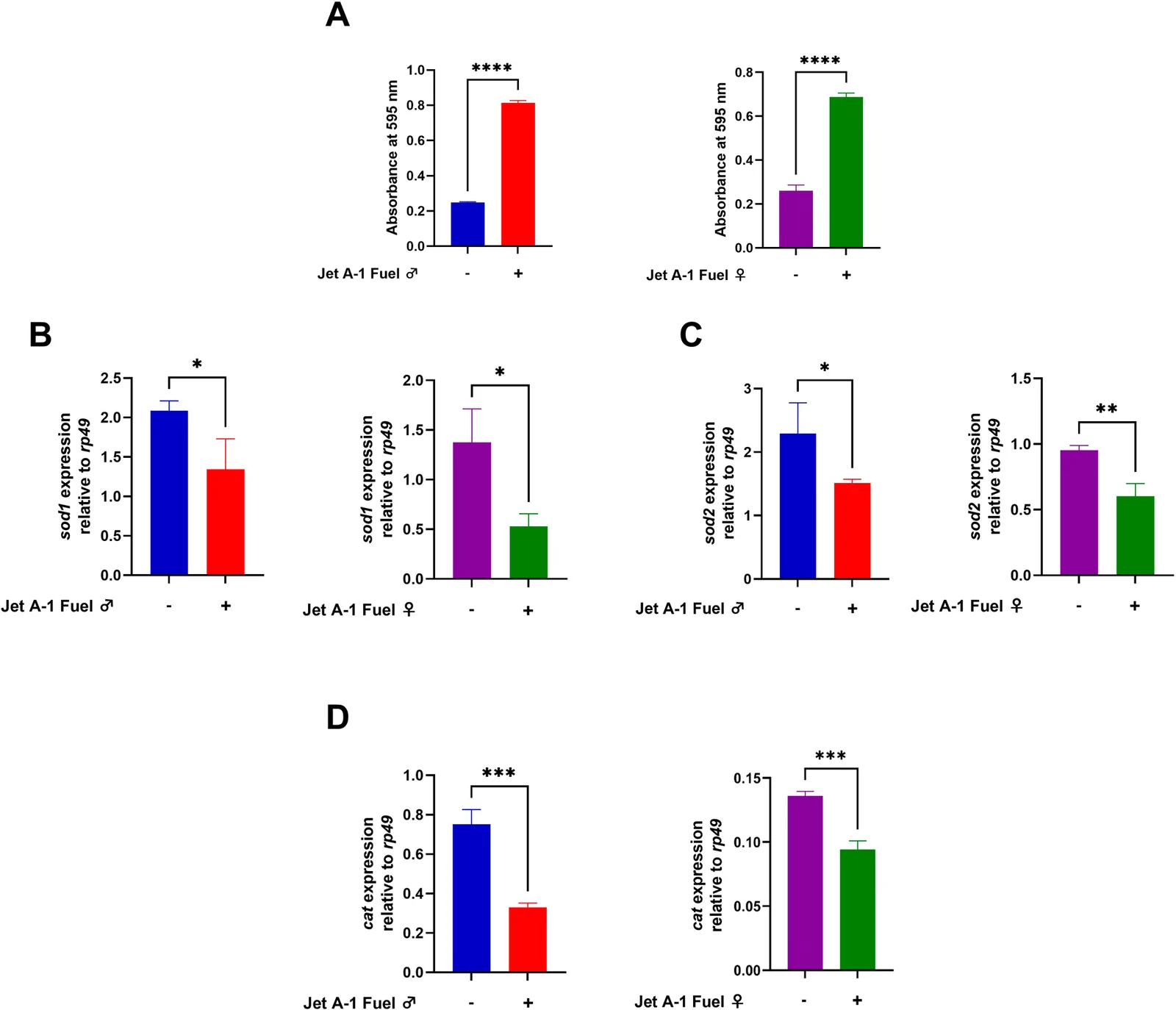
Jet A-1 fuel exposure elevated reactive oxygen species (ROS) levels in male and female *D. melanogaster* (A), as indicated by increased absorbance at 595 nm. This oxidative stress was accompanied by significant alterations in the expression of endogenous antioxidant genes, including *sod1* (B), *sod2* (C), and *cat* (D). Statistical significance is indicated as *p < 0.05, **p < 0.01, ***p < 0.001, and ****p < 0.0001.

### 3.4 Chemical Characterization of Jet A-1 Fuel

In order to evaluate the chemical compositions of the jet fuel used in this study, we utilize GC-MS. The analysis of the AVTUR Jet A-1 sample revealed a diverse hydrocarbon mixture, predominantly comprising C8–C16 compounds characteristic of kerosene-based aviation turbine fuels.^41,42^ The total ion chromatogram displayed multiple peaks eluting between 3 and 17 min, with major components identified via spectral library matches and evaluated through their fragmentation patterns (Table 2).

**Table 2:** Chemical Compositions of A-1 Jet Fuel Examined Using GC-MS

Compound	Molecular Formula	Retention Time (min)	Relative Concentration (%)	Base Peak *m*/*z*	Molecular *m*/*z*
Pentane, 2,2,3,4-tetramethyl-	C9H20	3.067	0.01	57	128
Heptane, 3-methyl-	C8H18	3.127	0.03	57	114
Cyclohexane, 1,3-dimethyl-, cis-	C8H16	3.308	0.19	97	112
Heptane, 2,6-dimethyl-	C9H20	4.12	0.03	57	128
Cyclohexane, 1,1,3-trimethyl-	C9H18	4.328	0.26	111	126
Cyclohexane, 1,3,5-trimethyl-	C9H18	4.644	0.15	69	126
Nonane, 4,5-dimethyl-	C11H24	4.834	0.16	57	156
Octane, 3-methyl-	C9H20	4.966	0.39	57	128
1-Ethyl-4-methylcyclohexane	C9H18	5.384	0.51	97	126
Nonane	C9H20	5.576	1.12	57	128
4-Methylnonane	C10H22	6.192	0.71	57	142
Benzene, 1-ethyl-2-methyl-	C9H12	7.031	0.99	105	120
Nonane, 3-methyl-	C10H22	7.105	0.57	57	142
Decane	C10H22	7.495	1.8	57	142
Decane, 4-methyl-	C11H24	7.946	2.71	71	156
Undecane	C11H24	9.283	5.12	57	156
2-Methyldecalin	C11H20	9.601	0.91	81	152
Cyclohexane, pentyl-	C11H22	9.892	2.72	83	154
Octane, 4-ethyl-	C10H22	10.139	2.37	57	142
Undecane, 2-methyl-	C12H26	10.303	1.25	57	170
Dodecane	C12H26	10.898	4.55	57	170
Undecane, 2,6-dimethyl-	C13H28	11.07	2.56	57	184
Dodecane, 4-methyl-	C13H28	11.77	0.81	71	184
Dodecane, 2-methyl-	C13H28	11.855	1	57	184
Dodecane, 4,6-dimethyl-	C14H30	11.963	2.59	57	198
Naphthalene, 1,2,3,4-tetrahydro-6-methyl-	C11H14	12.081	1.02	131	146
Tridecane	C13H28	12.413	4.77	57	184
3-Methyldodecane	C13H28	12.63	0.71	57	184
Naphthalene, 1,2,3,4-tetrahydro-2,6-dimethyl-	C12H16	12.887	1.4	145	160
Tridecane, 4-methyl-	C14H30	13.234	0.49	71	198
Tridecane, 2-methyl-	C14H30	13.314	0.73	57	198
Hexadecane, 2,6,10,14-tetramethyl-	C20H42	13.466	1.73	57	282
Decahydro-1,1,4a,5,6-pentamethylnaphthalene	C15H28	13.575	0.54	193	208
Tetradecane	C14H30	13.834	2.33	57	198
Amorphene-A	C15H28	14.286	0.49	109	208
Decahydro-1,1,4a,5,6-pentamethylnaphthalene	C15H28	14.34	0.43	193	208
2,6,10-Trimethyltridecane	C16H34	14.64	0.99	57	226
2-N-butyl-3-*n*-hexyldecahydronaphthalene	C20H38	14.847	0.61	57	278
Pentadecane	C15H32	15.204	1.12	57	212
Hexadecane	C16H34	16.552	0.07	57	226

Straight chained alkanes contributed 20.88% of the relative concentration among the major identified peaks. This includes undecane (5.12%; RT 9.283 min), tridecane (4.77%; RT 12.413 min), dodecane (4.55%; RT 10.898 min), tetradecane (2.33%; RT 13.834 min), pentadecane (1.12%; RT 15.204 min), decane (1.80%; RT 7.495 min), and nonane (1.12%; RT 5.576 min), which are commonly reported in jet fuel samples.^43,44^ Branched alkanes are also identified, representing 19.84%, with prominent compounds such as 4-methyldecane (2.71%; RT 7.946 min), 4,6-dimethyldodecane (2.59%; RT 11.963 min), 2,6-dimethylundecane (2.56%; RT 11.070 min), 4-ethyloctane (2.37%; RT 10.139 min), pristane (2,6,10,14-tetramethylhexadecane; 1.73%; RT 13.466 min), and various other methyl-substituted isomers. Cycloalkanes comprised 5.23%, dominated by pentylcyclohexane (2.72%; RT 9.892 min), alongside trimethylcyclohexanes, decalin, and sesquiterpene derivatives. Aromatic hydrocarbons were present at lower levels in the profile, including 1-ethyl-2-methylbenzene (0.99%; RT 7.031 min) and decahydro-pentamethylnaphthalene (0.54%; RT 13.575 min), though minor peaks indicated additional alkylbenzenes such as xylenes. More specific, naphthalene derivatives such as 1,2,3,4-tetrahydro-6-methylnaphthalene (1.02%; RT 12.081 min) and 1,2,3,4-tetrahydro-2,6-dimethylnaphthalene (1.40%; RT 12.887 min) are also detected, totaling approximately 2.42% relative area.

Upon careful examination on the mass fragmentation pattern of both methyl naphthalene derivatives, we found that the base peak *m*/*z* 118 arising from *m*/*z* 146 and 160 indicate the loss of side chain (one or two CH_4_^+^ ions 14–28 Da). The mass spectrum pattern (Figure 5) does not indicate the fragmentation of unsubstituted naphthalene but clearly fits a methyl-tetralin structure, also reported in previous studies.^45–47^ To further verify the presence of naphthalene-related compounds in the Jet A-1 sample, a targeted UFLC analysis was performed using an external calibration method (Supporting Information, Figures S3–S4 and Tables S1–S2). The calibration curve exhibited excellent linearity over the concentration range of 1.25–20 ppm (R^2^ = 0.9973), indicating reliable quantification of naphthalene. Analysis of the Jet A-1 sample revealed a chromatographic peak at a retention time of approximately 4.15 min, corresponding to the naphthalene standard. Quantitative analysis indicated a naphthalene concentration ranging from 20.79 to 23.20 ppm in the diluted sample, corresponding to approximately 10.40–11.60 ppm in the original Jet A-1 fuel sample. However, considering the relatively low concentration of naphthalene compared with the overall hydrocarbon composition of Jet A-1, the pronounced toxicity observed in the biological assays is unlikely to be attributable solely to naphthalene and more likely reflects the combined effects of multiple fuel-derived hydrocarbons.

**Figure 5:**
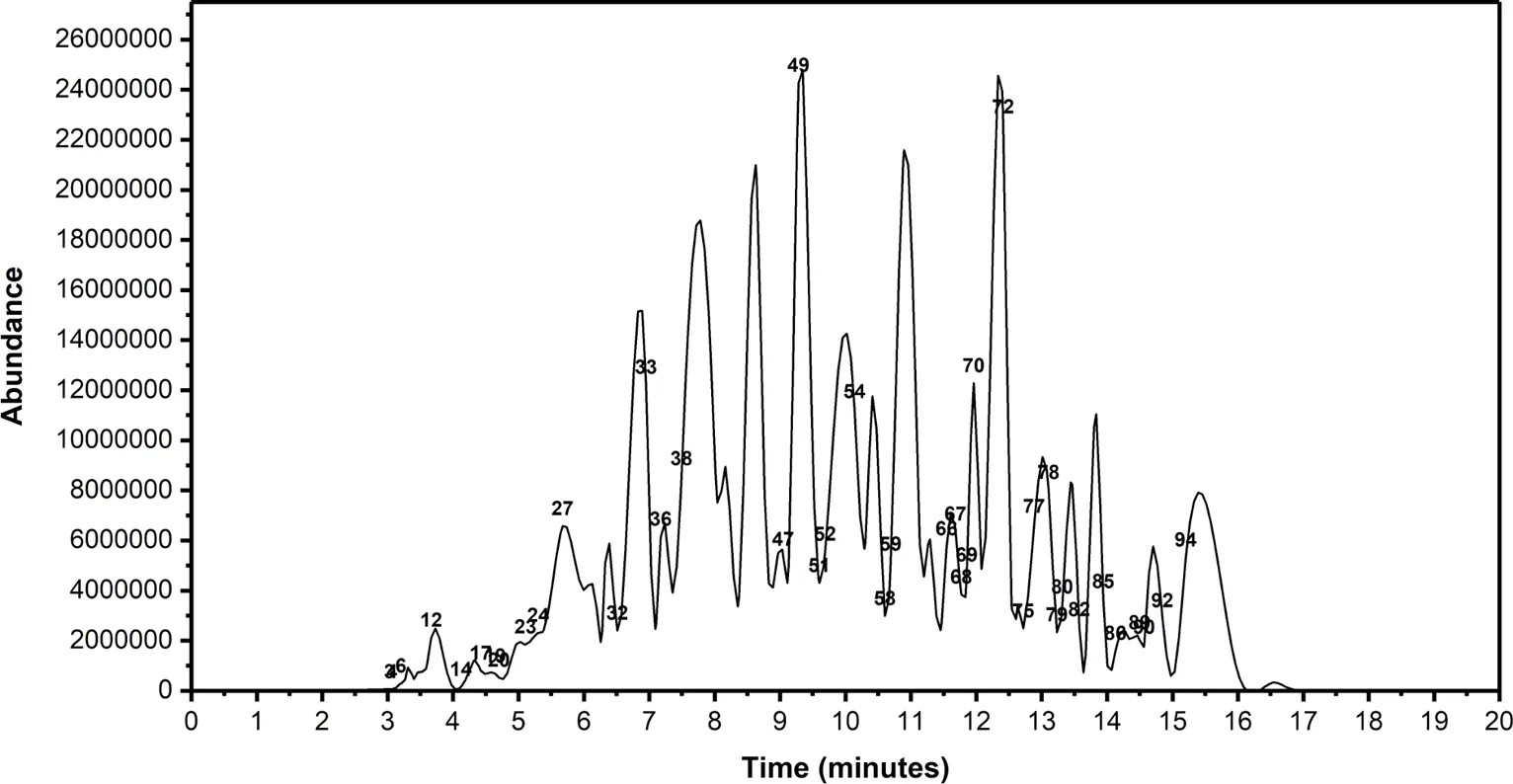
GC-MS spectra of Jet A-1 fuel.

### 3.5 Prediction of Candidate Targets and Construction of PPI Network

Matching the candidate targets of toxic compounds and the potential targets of neurotoxicity, to investigate the underlying molecular mechanisms of neurotoxicity induced by Avtur-A1, target screening was conducted followed by protein–protein interaction (PPI) network analysis. A total of 329 targets associated with Jet A-1 Fuel and 2289 targets linked to neurotoxicity were identified. The intersection of these two data sets yielded 129 common targets (Figure 6A), indicating a significant overlap that likely represents key molecular mediators involved in Jet A-1 Fuel-induced neurotoxicity. These common targets were then used to build an initial PPI network. The generated PPI network (Figure 6A) exhibited a high density of interactions among the selected targets, reflecting extensive protein–protein connectivity that may play a critical role in the development of neurotoxicity. This pronounced network density underscores the intricate biological complexity and multifaceted impact of Jet A-1 Fuel on neuronal systems. Moreover, the highly interconnected proteins likely represent key signaling hubs that govern the regulatory mechanisms underlying neurotoxic responses.

**Figure 6:**
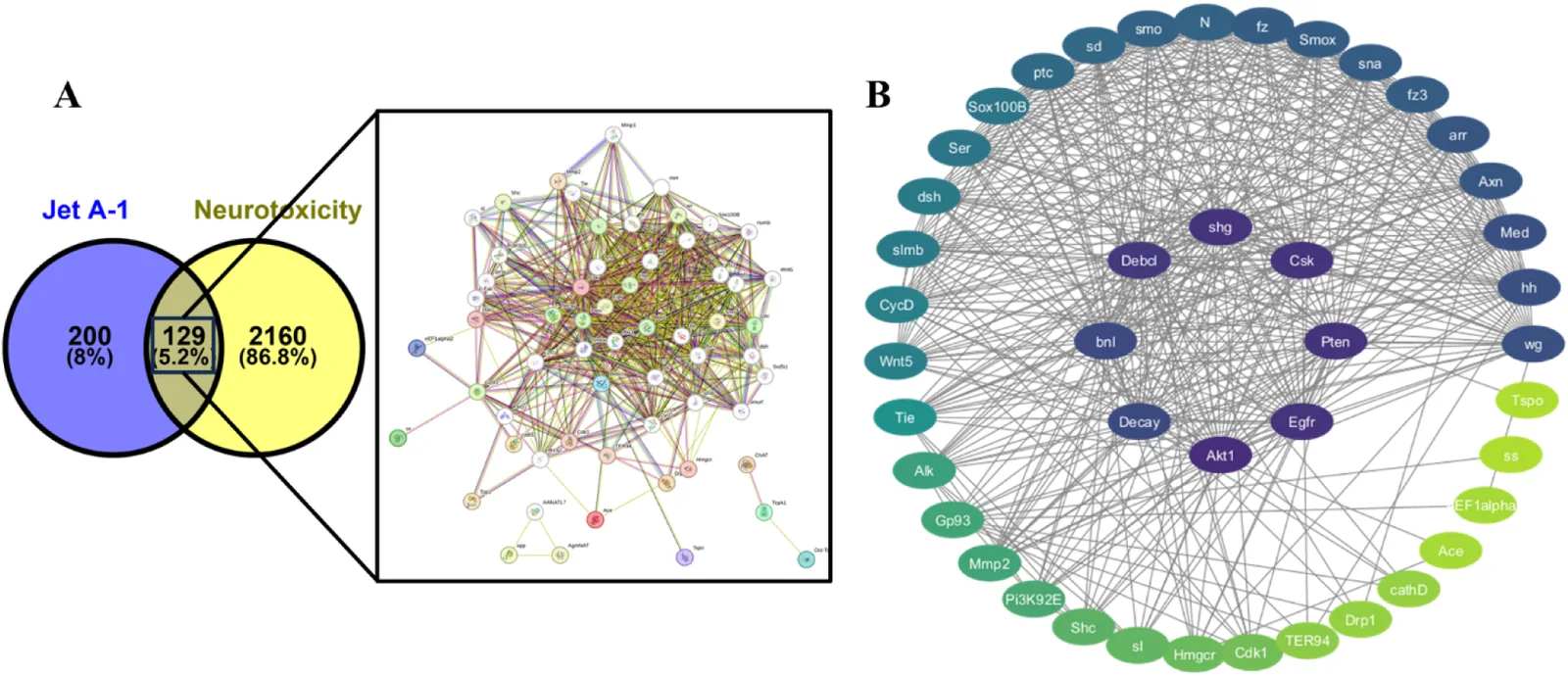
Target screening and establishment of PPI network. (A) Potential targets of neurotoxicity caused by Jet A-1 fuel and PPI network. (B) PPI network of neurotoxicity targets caused by Jet A-1 fuel. The color of the node is marked from purple to light green according to the degree value in descending order.

The top eight were selected (center) as the core targets based on the ranking of Degree value, followed by AKT1, EGFR, Pten, CSK, shg, Debcl, bnl, and Decay. The visualization results are shown in Figure 6B.

Based on the network pharmacology analysis, the findings suggest that compounds Jet A-1 may play a crucial role in the mechanisms of neurotoxicity associated with exposure to Jet A-1 Fuel. Naphthalene derivatives, a major component of jet fuels such as Jet A-1 Fuel, were identified in this analysis as a compound with the potential to disrupt the nervous system through several molecular pathways predicted from the interactions of related gene and protein targets.

### 3.6 GO Enrichment and KEGG Pathway Analysis

Analysis of 129 target gene interactions using the STRING database revealed extensive involvement in various biological processes and key signaling pathways. Gene Ontology (GO) analysis identified a total of 561 functional items categorized into three main groups: biological process, cellular component, and molecular function. Within key biological processes, particularly those involved in the regulation of locomotion, cell migration, cell death, neurogenesis, and neuronal development. These highly enriched processes indicate that the gene set is strongly associated with cellular dynamics, neuronal differentiation, and the modulation of apoptotic responses. Within the Cellular Component category, enriched terms were predominantly associated with the plasma membrane, cell periphery, cytoplasm, and membrane-bounded organelles, suggesting that the majority of target genes function within membrane-associated compartments where signal transduction and regulatory interactions occur. In the Molecular Function category, enrichment of signaling receptor binding, protein binding, enzyme binding, and nucleotide binding functions highlights the central role of ligand–receptor interactions and intracellular regulatory mechanisms in mediating the observed biological responses (Figure 7A).

**Figure 7:**
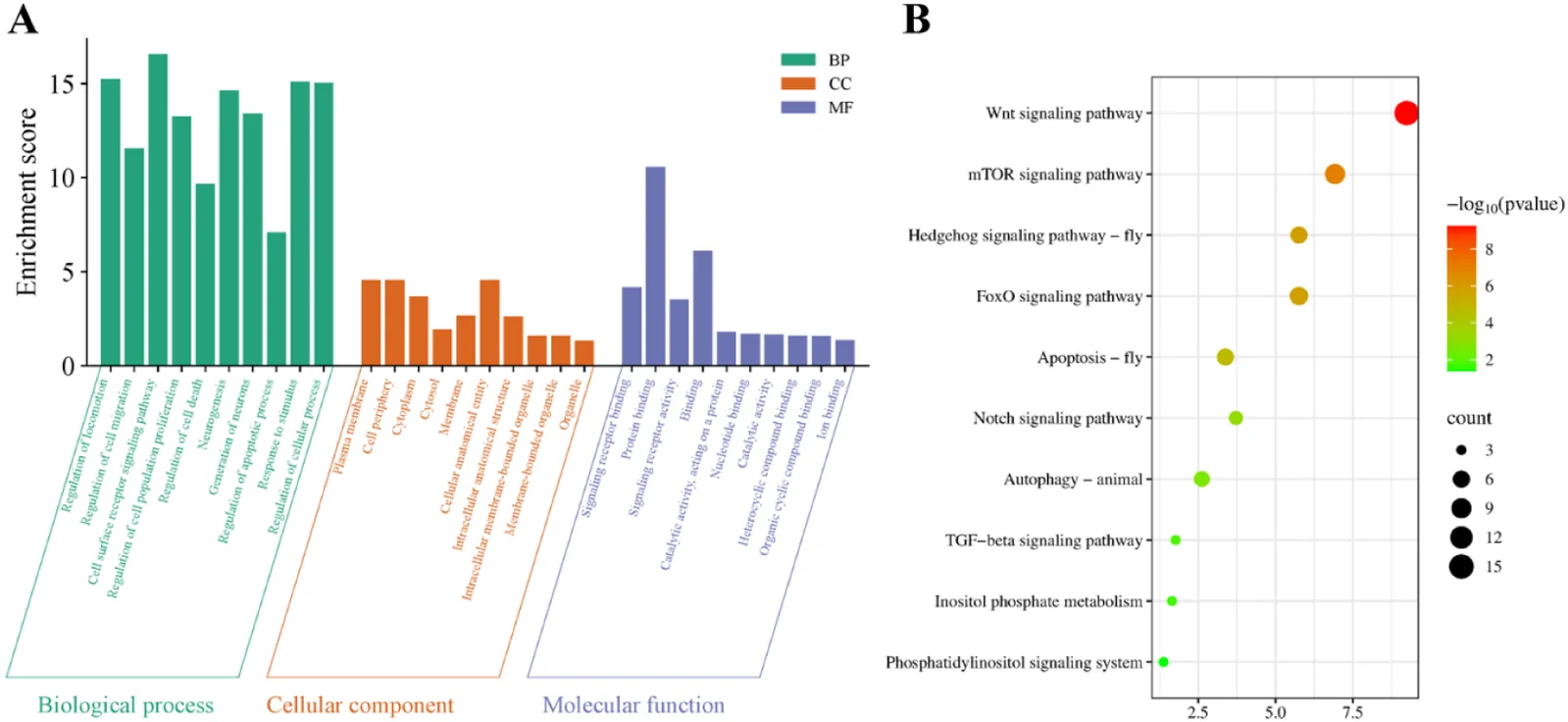
GO enrichment and KEGG pathway analysis. (A) The bar plot diagram of GO enrichment analysis of 129 intersection targets, including significant enrichment terms of biological process (BP), cellular component (CC), and molecular function (MF). (B) The bubble plot diagram of KEGG pathway enrichment analysis of 129 intersection targets (top 10).

The KEGG pathway enrichment analysis further supported these findings by identifying several highly significant pathways, including Wnt, mTOR, and Hedgehog signaling pathways, followed by FoxO signaling, apoptosis, Notch signaling, and autophagy. These pathways are well-known to regulate neuronal development, cellular homeostasis, survival mechanisms, and stress responses (Figure 7B). The enrichment of inositol phosphate metabolism and phosphatidylinositol signaling pathways additionally underscores the involvement of membrane-associated signaling cascades, particularly those regulating intracellular calcium flux and phospholipid-mediated signal transduction. Collectively, the combined GO and KEGG enrichment results suggest that the analyzed gene set is primarily involved in the modulation of neuronal function, membrane-associated signaling, and survival-related cellular processes. The convergence of pathways governing neurogenesis, stress adaptation, and programmed cell death indicates that the biological responses observed in this study likely arise from integrated alterations in neuronal regulatory mechanisms and membrane-dependent signaling networks.

### 3.7 Molecular Docking and Receptor–Ligand Interaction Analysis

To elucidate the molecular basis of Jet A-1 Fuel associated neurotoxicity, 39 representative toxic constituents detected in Jet A-1 fuel (Table 3) were subjected to molecular docking analysis against key protein targets (Figure 7). Eight core proteins AKT1, EGFR, PTEN, CSK, shg (DE-cadherin), Debcl, bnl, and Decay were selected based on their high degree values in the protein–protein interaction (PPI) network, indicating their central regulatory roles in neuronal survival, stress response, and apoptosis.

**Table 3:** Result of Molecular Docking between Jet A-1 Compounds and Key Toxic Substances

	Binding Energy (kcal/mol)					
Compound	**AKT1**	**EGFR**	**PTEN**	**CSK**	**SHG**	**DECAY**
Pentane, 2,2,3,4-tetramethyl-	–5.2	–4.5	–4.9	–4.6	–4.0	–3.7
Heptane, 3-methyl-	–4.6	–3.8	–4.4	–4.2	–4.1	–3.9
Cyclohexane, 1,3-dimethyl-, cis-	–5.7	–4.4	–5.2	–4.9	–4.3	–3.8
Heptane, 2,6-dimethyl-	–5.2	–4.1	–4.6	–4.7	–4.1	–3.7
Cyclohexane, 1,1,3-trimethyl-	–5.7	–4.8	–5.0	–5.1	–4.6	–4.1
Cyclohexane, 1,3,5-trimethyl-	–5.8	–4.7	–4.9	–5.1	–4.1	–4.1
Nonane, 4,5-dimethyl-	–5.5	–4.6	–4.4	–5.1	–4.4	–4.1
Octane, 3-methyl-	–5.0	–4.1	–4.4	–4.7	–4.2	–4.1
1-Ethyl-4-methylcyclohexane	–5.9	–4.9	–5.2	–5.2	–4.7	–4.4
Nonane	–4.9	–4.1	–4.2	–4.5	–4.1	–3.7
4-Methylnonane	–5.3	–4.5	–4.5	–4.7	–4.3	–3.9
Benzene, 1-ethyl-2-methyl-	–6.2	–5.0	–5.1	–5.3	–4.8	–4.2
Nonane, 3-methyl-	–5.2	–4.4	–3.9	–4.9	–4.2	–4.0
Decane	–5.2	–4.2	–3.9	–4.6	–4.1	–3.7
Decane, 4-methyl-	–5.5	–4.7	–4.5	–4.9	–4.3	–3.8
Undecane	–5.3	–4.4	–4.5	–4.8	–4.3	–3.6
2-Methyldecalin	–7.2	–5.8	–5.2	–6.4	–5.5	–4.6
Cyclohexane, pentyl-	–6.2	–5.3	–4.4	–5.7	–5.4	–4.9
Octane, 4-ethyl-	–5.1	–4.0	–4.6	–4.8	–4.2	–3.8
Undecane, 2-methyl-	–5.8	–4.7	–4.3	–5.0	–4.4	–4.0
Dodecane	–5.4	–4.0	–3.7	–4.8	–4.4	–4.0
Undecane, 2,6-dimethyl-	–6.1	–4.8	–4.2	–5.2	–4.6	–4.1
Dodecane, 4-methyl-	–5.9	–4.9	–4.1	–5.2	–4.2	–3.9
Dodecane, 2-methyl-	–5.8	–4.8	–4.6	–5.1	–4.6	–3.8
Dodecane, 4,6-dimethyl-	–6.3	–5.0	–4.2	–5.4	–4.4	–3.9
Naphthalene, 1,2,3,4-tetrahydro-6-methyl-	–7.7	–6.0	–5.5	–6.5	–6.2	–4.7
Tridecane	–5.6	–4.6	–3.9	–4.9	–4.5	–3.9
3-Methyldodecane	–5.9	–4.7	–4.3	–5.1	–4.2	–3.7
Naphthalene, 1,2,3,4-tetrahydro-2,6-dimethyl-	–8.1	–6.2	–5.8	–6.9	–5.8	–4.9
Tridecane, 4-methyl-	–5.9	–4.7	–4.2	–5.1	–4.1	–4.1
Tridecane, 2-methyl-	–6.0	–4.9	–4.4	–4.8	–4.8	–3.8
Hexadecane, 2,6,10,14-tetramethyl-	–7.0	–5.6	–4.7	–5.9	–4.7	–4.7
Decahydro-1,1,4a,5,6-pentamethylnaphthalene	–7.6	–6.7	–5.6	–7.3	–4.9	–4.8
Tetradecane	–5.7	–4.8	–4.1	–5.1	–4.3	–3.4
Amorphene-A	–8.0	–6.5	–5.7	–6.9	–5.9	–5.0
2,6,10-Trimethyltridecane	–6.5	–5.3	–4.7	–5.6	–4.4	–3.7
2-N-butyl-3-*n*-hexyldecahydronaphthalene	–8.4	–7.0	–5.0	–6.5	–5.8	–4.9
Pentadecane	–5.9	–4.7	–4.1	–5.1	–4.2	–3.7
Hexadecane	–5.9	–4.6	–4.1	–4.9	–4.2	–3.7

As shown in the binding energy heatmap, AKT1 exhibited the strongest interactions across multiple ligands, with binding energies reaching −8.4 kcal/mol, suggesting that AKT1 is a highly susceptible target for Jet A-1 toxic compounds. Given the essential role of AKT1 in maintaining neuronal survival and mitochondrial integrity, such strong binding interactions are consistent with a potential inhibitory modulation that may attenuate downstream pro-survival signaling. Similarly, EGFR and CSK, both kinase-related signaling regulators, displayed consistently negative binding energies (approximately −6.0 to −7.3 kcal/mol), indicating that ligand binding may interfere with growth factor and Src-related signaling pathways that are critical for neuronal homeostasis and adaptive stress responses. PTEN, a key phosphatase regulating the PI3K-AKT axis, also showed moderate to strong binding affinities, suggesting that its enzymatic or regulatory function may be negatively modulated. Concurrent inhibition of AKT1 and PTEN is expected to cause dysregulation of survival signaling rather than balanced pathway control, thereby sensitizing neurons to oxidative and apoptotic insults. In contrast, shg (DE-cadherin) and bnl, which are nonenzymatic structural and signaling proteins, respectively, exhibited binding energies indicative of modulatory rather than inhibitory interactions. Ligand binding to shg may perturb cell–cell adhesion and tissue integrity, while interaction with bnl may interfere with FGF–FGFR recognition and downstream neuroprotective signaling. The mitochondrial apoptosis regulator Debcl showed notable ligand affinity, supporting the notion that Jet A-1 constituents may directly influence intrinsic apoptotic pathways. Additionally, Decay, a protein involved in mRNA turnover and stress-related gene regulation, demonstrated consistent binding, suggesting potential disruption of post-transcriptional stress adaptation mechanisms. Collectively, these results indicate that Jet A-1–derived toxic compounds do not act through a single molecular target, but rather exert multitarget inhibitory and modulatory effects that converge on survival signaling suppression, redox imbalance, mitochondrial dysfunction, and activation of apoptosis.^13^

Overall, the heatmap demonstrates that Jet A-1 fuel constituents interact with multiple core proteins with varying affinities (Figure 8), highlighting a multitarget mode of action. The convergence of strong inhibitory-leaning interactions on kinases such as AKT1, EGFR, and CSK, together with modulatory effects on PTEN, shg, and Decay, supports a mechanistic framework in which coordinated disruption of survival signaling, stress adaptation, and regulatory homeostasis contributes to Jet A-1 associated neurotoxicity, rather than effects mediated by a single dominant molecular target.

**Figure 8:**
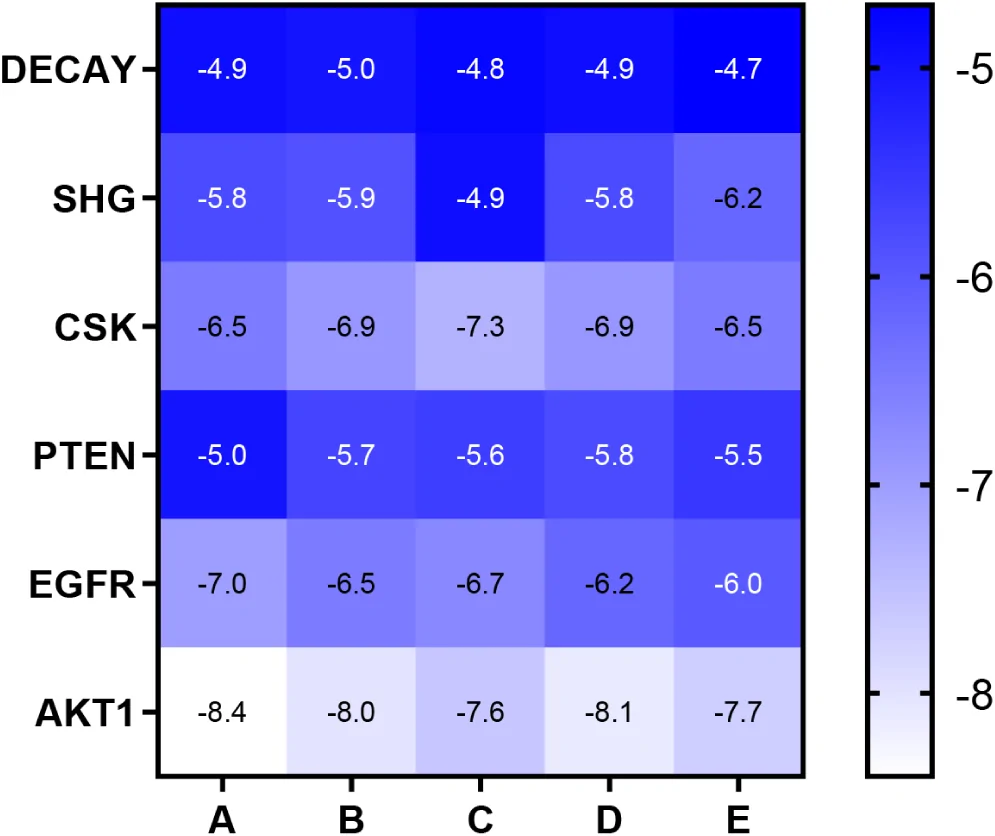
Heatmap of binding energy between key toxic substances, the degree value ranking, and core targets of (A) 2-*N*-butyl-3-*n*-hexyldecahydronaphthalene. (B) Amorphene-A. (C) Decahydro-1,1,4a,5,6-pentamethylnaphthalene. (D) Naphthalene, 1,2,3,4-tetrahydro-2,6-dimethyl-. (E) Naphthalene, 1,2,3,4-tetrahydro-6-methyl-.

Excessive or prolonged exposure to naphthalene is known to increase neurotoxic risk, as its metabolites generate reactive oxygen species (ROS), induce oxidative stress, impair mitochondrial stability, and trigger apoptosis, particularly through modulation of AKT1, FOXO, PTEN, and SMOX signaling pathways.

Figure 9 presents the three-dimensional (3D) representations of the molecular interactions between Jet A-9 compounds and the key target proteins identified in this study. Figure 9A illustrates the interaction between 2-*n*-butyl-3-*n*-hexyldecahydronaphthalene and AKT1, revealing multiple contact points involving residues such as LYS268, TYR272, LEU210, TRP80, and VAL270. These residues are located within or adjacent to the ATP-binding and catalytic regions of AKT1, which have been reported as critical for kinase activity and signal transduction.^48^ The occupation of this region by 2-*n*-butyl-3-*n*-hexyldecahydronaphthalene supports its potential role as a negative modulator of AKT1 activity, thereby weakening pro-survival signaling pathways. In Figure 9B, the interaction between 2-*n*-butyl-3-*n*-hexyldecahydronaphthalene and EGFR is shown to involve key residues including LYS721, VAL702, LEU764, and ALA719, mediated through π-cation, π-alkyl, and hydrophobic interactions. These residues are well recognized as components of the EGFR kinase domain and ATP-binding pocket.^49^ Therefore, the observed binding mode suggests that 2-*n*-butyl-3-*n*-hexyldecahydronaphthalene may function as a potential inhibitor or negative modulator of EGFR catalytic activity, leading to attenuation of EGFR-mediated pro-survival signaling and contributing to neuronal vulnerability under toxic exposure conditions. In Figure 9C, the PTEN-Naphthalene,1,2,3,4-tetrahydro-2,6-dimethyl interaction is characterized by hydrogen bonding and π-alkyl contacts with LYS128, residues situated close to the phosphatase catalytic core and known to contribute to shaping the active-site cleft.^50^ Binding of Naphthalene,1,2,3,4-tetrahydro-2,6-dimethyl in this region may interfere with substrate recognition or catalytic efficiency, resulting in dysregulation of PTEN activity and subsequent perturbation of the PI3K-AKT signaling axis. Figure 9D depicts the binding of decahydro-1,1,4a,5,6-pentamethylnaphthalene to CSK, a key regulator of Src family kinases including VAL209, ALA220, LYS222, and LEU321. The ligand occupies a hydrophobic pocket adjacent to the regulatory domain, implying possible interference with CSK-Src interactions rather than direct catalytic inhibition.^51^ Altered CSK regulation may dysregulate downstream signaling involved in neuronal stress responses. In Figure 9E, naphthalene1,2,3,4-tetrahydro-6-methyl- binds to SHG (DE-cadherin) at a region associated with protein–protein interactions. As SHG is a structural and adhesion protein rather than an enzyme, this interaction is best interpreted as modulatory, potentially affecting cell–cell adhesion stability and tissue integrity under toxic stress conditions.^52,53^ Figure 9F depicts the binding interface between Amorphene-A and Decay (DC55), highlighting a combination of hydrogen bonding and hydrophobic interactions primarily involving ILE82 and LEU77. Although experimentally validated binding sites for Decay remain limited, the docking results suggest that Amorphene-A occupies a structurally relevant region that may influence Decay conformation.^54^ Such modulation could interfere with its regulatory role in apoptosis and stress-related signaling pathways.

**Figure 9:**
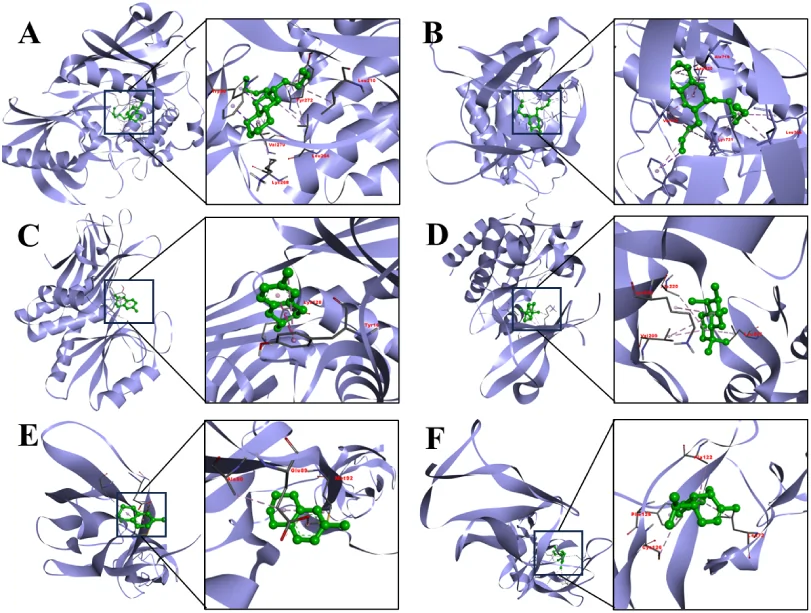
Interactions between Jet A-1 fuel compounds and key proteins: (A) 2-*n*-butyl-3-*n*-hexyldecahydronaphthalene-AKT1, (B) 2-*n*-butyl-3-*n*-hexyldecahydronaphthalene-EGFR, (C) naphthalene, 1,2,3,4-tetrahydro-2,6-dimethyl-PTEN, (D) decahydro-1,1,4a,5,6-pentamethylnaphthalene-CSK, (E) naphthalene, 1,2,3,4-tetrahydro-6-methyl-SHG, and (F) amorphene-A-DECAY.

Taken together, the findings presented in Figure 9 support a multitarget molecular mechanism through which Jet A-1 fuel components may perturb neuronal homeostasis. The identified interactions involve a diverse range of molecular targets, including kinases, phosphatases, regulatory proteins, and structural proteins, with predominantly hydrophobic interactions contributing to ligand–protein binding. The combined inhibitory effects predicted for key signaling regulators, including AKT1, EGFR, PTEN, and CSK, together with potential perturbation of SHG and Decay, suggest a broader disruption of cellular survival and signaling networks. Such coordinated molecular disturbances may impair pro-survival signaling, increase cellular susceptibility to oxidative stress, and ultimately facilitate apoptotic processes, providing a plausible mechanistic basis for Jet A-1-associated neurotoxicity, particularly following prolonged or excessive exposure.

Align with the in vivo findings, we further propose that Jet A-1 exposure induces mitochondrial oxidative stress, characterized by excessive ROS generation and compromised endogenous antioxidant defenses, potentially involving dysregulation of *sod* and *cat* expression (Figure 10). The convergence of oxidative stress, disrupted survival signaling, mitochondrial dysfunction, and cholinergic dysregulation may consequently contribute to the reduced survival and locomotor activity observed following Jet A-1 exposure. Collectively, these findings suggest that Jet A-1-induced neurotoxicity may arise from the integrated disruption of multiple interconnected molecular and cellular pathways rather than from the modulation of a single molecular target.

**Figure 10:**
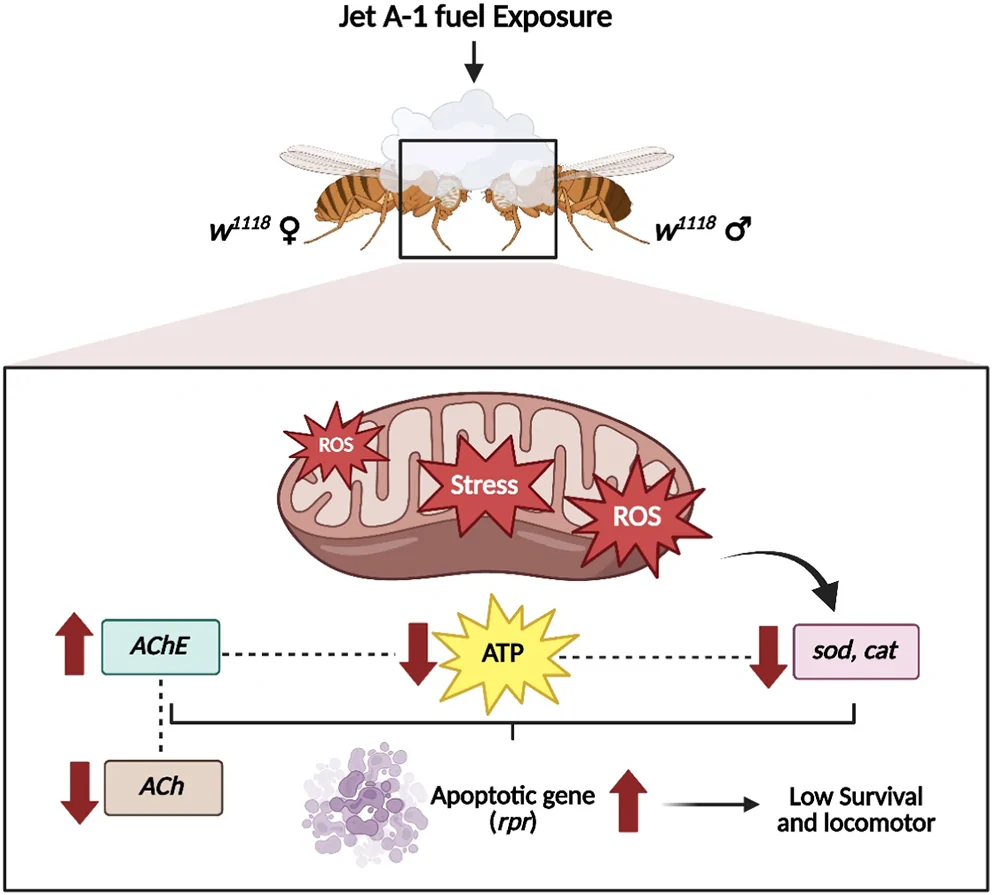
Proposed mechanism of Jet A-1 fuel-induced toxicity in *Drosophila melanogaster*. Exposure to Jet A-1 increases mitochondrial reactive oxygen species (ROS) production, leading to disruption of the electron transport chain, reduced ATP synthesis, and elevated cellular stress. Created in BioRender. https://BioRender.com/db6jhhy.

## 4 Conclusion

This study demonstrates that exposure to Jet A-1 induces acute neurotoxic effects in *D. melanogaster* through multiple molecular mechanisms. The findings reveal that naphthalene derivatives, which are major components of jet fuels such as Jet A-1, contribute tooxidative stress, increased ROS production, and activation of apoptotic signaling. In addition, Jet A-1 disrupts cholinergic function, as indicated by the upregulation of *AChE*, while impairing antioxidant defense mechanisms. These findings suggest that oxidative stress, disruption of cholinergic signaling, and apoptosis are among the central mechanisms underlying Jet A-1 induced neurotoxicity. Collectively, this study provides insights into the molecular basis of hydrocarbon fuel toxicity and supports the use of *Drosophila* as a model organism for investigating the neurotoxic effects of environmental contaminants.

## Supplementary Material



## Notes

Experimental procedures, survival assays of naphthalene and solvent controls (Figures S1–S2), UFLC calibration and chromatographic analyses (Figures S3–S4), and quantitative naphthalene data (Tables S1–S2) (PDF)
