# Supplementary material for: Dual In Vivo and In Silico Exploration of Jet A‑1 Fuel Toxicity
Source: ACS Omega. 2026 Sep 25;11(39):58507–19. doi: 10.1021/acsomega.5c10984 (PMC13647391; doi:10.1021/acsomega.5c10984)
Supplement: Supplementary file 1 [file ao5c10984_si_001.pdf]

Supporting Information

Dual In Vivo and In Silico Exploration of Jet A-1 Fuel Toxicity

Yuli Subiakto[1*], Yahdiana Harahap[1], Widya Hardiyanti[2,3], Budi Sumaryono[1], Habibie Habibie[4], Muh. Arfandy Gunawan[5], Tesia Aisyah Rahmania[1], Muhammad Raihan[6], Nadila Pratiwi Latada[2], Firzan Nainu[2,4*]

[1]Department of Military Pharmacy, Faculty of Military Pharmacy, Republic of Indonesia Defense University, Bogor 16815, Indonesia
[2]Unhas Fly Research Group, Faculty of Pharmacy, Hasanuddin University, Makassar 90245, Indonesia
[3]Program Study of Pharmacy, Faculty of Medicine and Health Sciences, Universitas Muhammadiyah Makassar, Makassar 90221, Indonesia
[4]Department of Pharmacy, Faculty of Pharmacy, Hasanuddin University, Makassar 90245, Indonesia
[5]Chemical Biology and Medicinal Chemistry Research Group, Faculty of Pharmacy, Hasanuddin University, Makassar 90245, Indonesia
[6]Department of Pharmaceutical Science and Technology, Faculty of Pharmacy, Hasanuddin University, Makassar 90245, Indonesia

***Corresponding authors:**

Yuli Subiakto
Department of Military Pharmacy, Faculty of Military Pharmacy, Republic of Indonesia Defense University, Bogor 16815, Indonesia
y.subiakto09@gmail.com

Firzan Nainu
Department of Pharmacy, Faculty of Pharmacy, Hasanuddin University, Makassar 90245, Indonesia
firzannainu@unhas.ac.id

Supporting Methods

1. *Preparation of Naphthalene Standard Solution Using Acetonitrile-Ethanol as Solvent*. A stock solution of naphthalene was prepared by accurately weighing 10 mg of naphthalene standard and dissolving it in acetonitrile to a final volume of 10 mL in a volumetric flask, yielding a concentration of 1000 ppm Subsequently, a working solution was prepared by transferring 0.11 mL of the 1000 ppm stock solution into a 10 mL volumetric flask. The solution was then diluted to volume with ethanol to obtain a final concentration of 11 ppm.
2. *Preparation of Naphthalene Standard Solution Using Dimethyl Sulfoxide (DMSO) as Solvent*. A stock solution of naphthalene was prepared by accurately weighing 10 mg of naphthalene standard and dissolving it in DMSO to a final volume of 10 mL in a volumetric flask, resulting in a concentration of 1000 ppm. For the preparation of the working solution, 0.11 mL of the 1000 ppm stock solution was transferred into a 10 mL volumetric flask and diluted to volume with DMSO to obtain a final concentration of 2.2 ppm.
3. *Survival Assay in Drosophila melanogaster Exposed to Jet A-1 Fuel*. A survival assay was conducted using adult *D. melanogaster*. Three-day-old male and female flies were collected separately and transferred into clean glass test tubes. For exposure to Jet A-1, DMSO, acetonitrile-ethanol, naphthalene dissolved in DMSO, and naphthalene dissolved in acetonitrile-ethanol, ten flies were placed into each test tube and sealed with a cotton plug. Subsequently, 500 µL of the respective test solution was applied to the upper surface of the cotton plug. The tubes were then tightly sealed with an additional stopper to prevent vapor leakage. Flies were monitored continuously until all individuals had died. Mortality was recorded at one-minute intervals. Each treatment was performed in triplicate and compared with an untreated control group.
4. *Determination of Naphthalene Calibration Curve Using Ultra Fast Liquid Chromatography (UFLC)*. The calibration curve of naphthalene was established using an UFLC system. A stock solution was prepared by accurately weighing 100 mg of naphthalene standard and dissolving it in a 10 mL volumetric flask with acetonitrile:water (70:30, v/v) to obtain a concentration of 10,000 ppm. The stock solution was subsequently diluted stepwise with the same solvent mixture to obtain a series of working standard solutions at concentrations of 20, 10, 5, 2.5, and 1.25 ppm. All prepared solutions were filtered through a 0.22 µm membrane filter prior to injection. Chromatographic analysis was performed using a reversed-phase C18 column (150 mm × 4.6 mm, 5 µm particle size). The mobile phase consisted of acetonitrile and water (70:30, v/v) under isocratic elution at a flow rate of 1.0 mL/min. The injection volume was 20 µL. Detection was carried out using a photodiode array (PDA) detector at 270 nm. Naphthalene was identified at a retention time of approximately 4.1 minutes. The calibration curve was constructed by plotting concentration versus integrated peak area, and linearity was evaluated using the correlation coefficient (R^2).
5. *Determination of Naphthalene Content in Jet-A1*. The quantification of naphthalene in the Jet-A1 sample was performed using an UFLC system. Briefly, 1 g of Jet-A1 was accurately weighed and transferred into a 10 mL volumetric flask, then diluted to volume with acetonitrile to obtain a solution with a concentration of 100,000 ppm (100 mg/mL). An aliquot of 1 mL of this solution was further transferred into a 10 mL volumetric flask and diluted to volume with

acetonitrile:water (70:30, v/v) to obtain a final concentration of 1,000 ppm. The sample solution was filtered through a 0.22 μm membrane filter prior to chromatographic analysis. Chromatographic separation was carried out using a reversed-phase C18 column (150 mm × 4.6 mm, 5 μm particle size) maintained at 40°C. The mobile phase consisted of acetonitrile and water (70:30, v/v) under isocratic elution at a flow rate of 1.0 mL/min. The injection volume was 20 μL. Detection was performed using a photodiode array (PDA) detector set at 270 nm. Naphthalene was identified by comparing its retention time with that of the reference standard (approximately 4.1 min). Quantification was performed using the regression equation obtained from the validated external calibration curve of naphthalene.

Supporting Figures

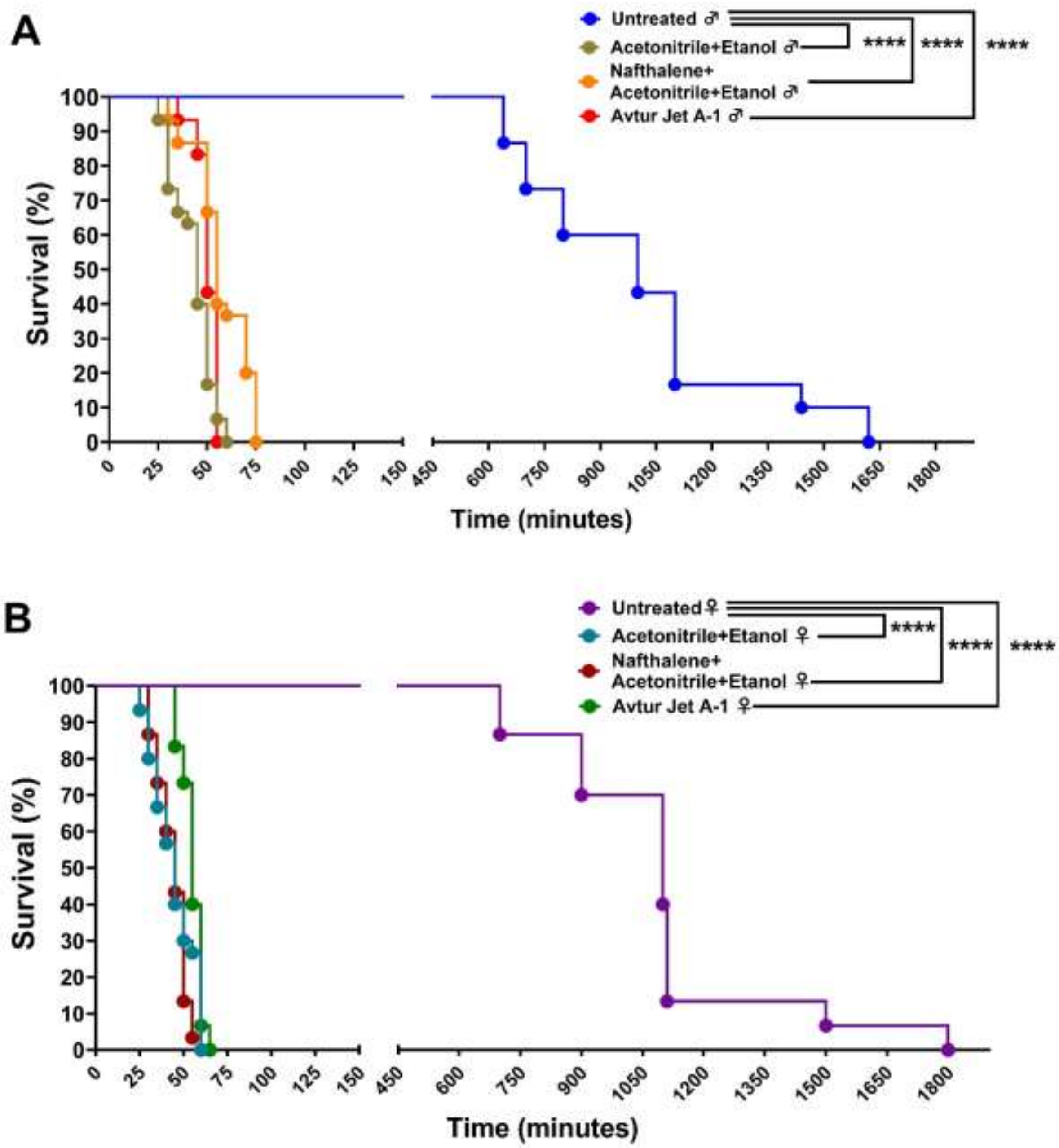

Figure S1. Survival curves of *Drosophila melanogaster* following inhalation exposure to different treatments using acetonitrile-ethanol as solvent. (A) Male flies (♂) and (B) female flies (♀). A significant reduction in survival was observed following inhalation exposure to the solvent control (acetonitrile + ethanol), naphthalene in combination with the solvent, and Jet A-1 fuel. Statistical significance is indicated as $^{****}p < 0.0001$.

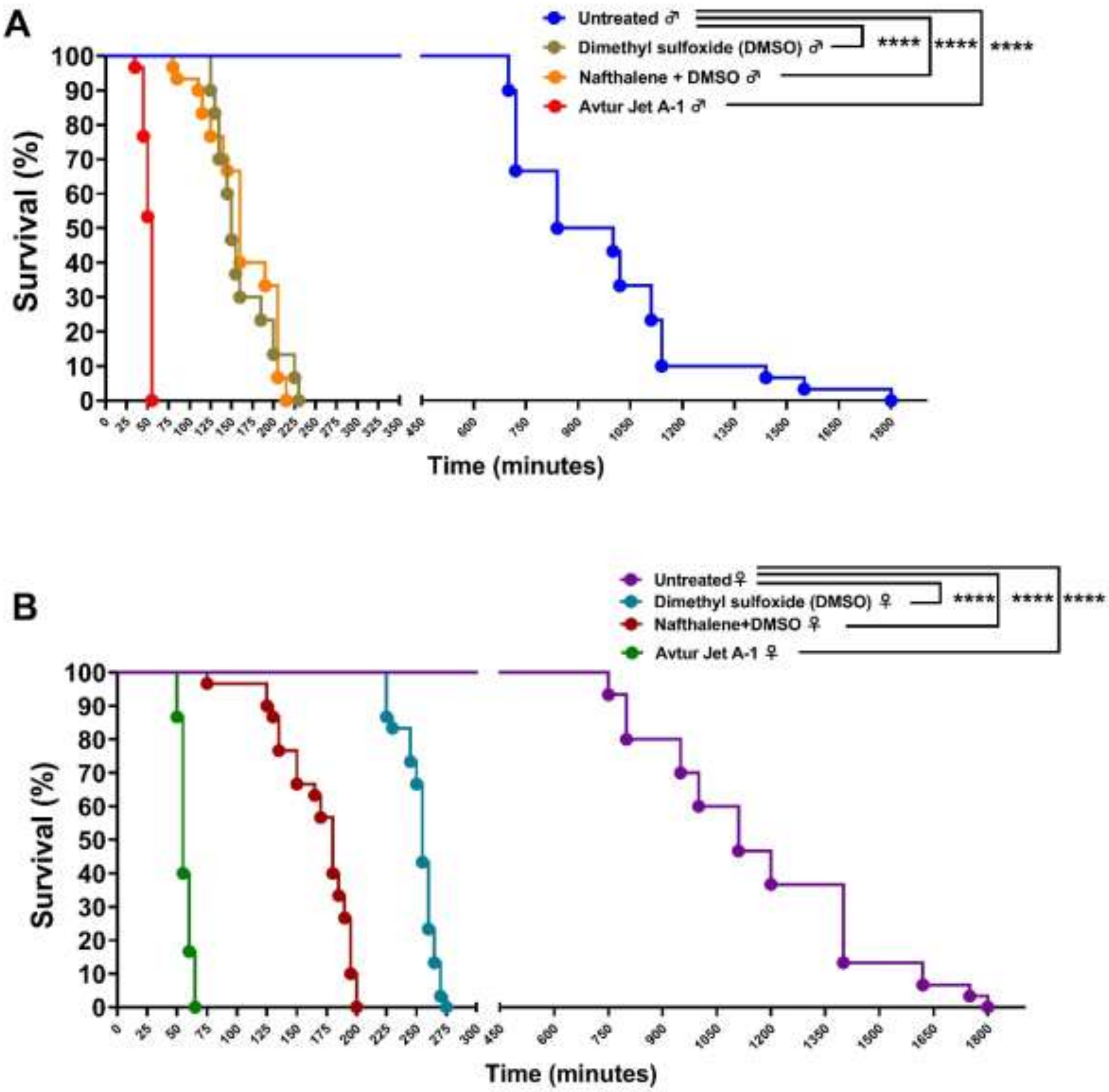

Figure S2. Survival curves of *Drosophila melanogaster* following inhalation exposure to different treatments using dimethyl sulfoxide (DMSO) as solvent. (A) Male flies (♂) and (B) female flies (♀). A significant reduction in survival was observed following inhalation exposure to the solvent control DMSO, naphthalene in combination with the solvent, and Jet A-1 fuel. Statistical significance is indicated as ****$p < 0.0001$.

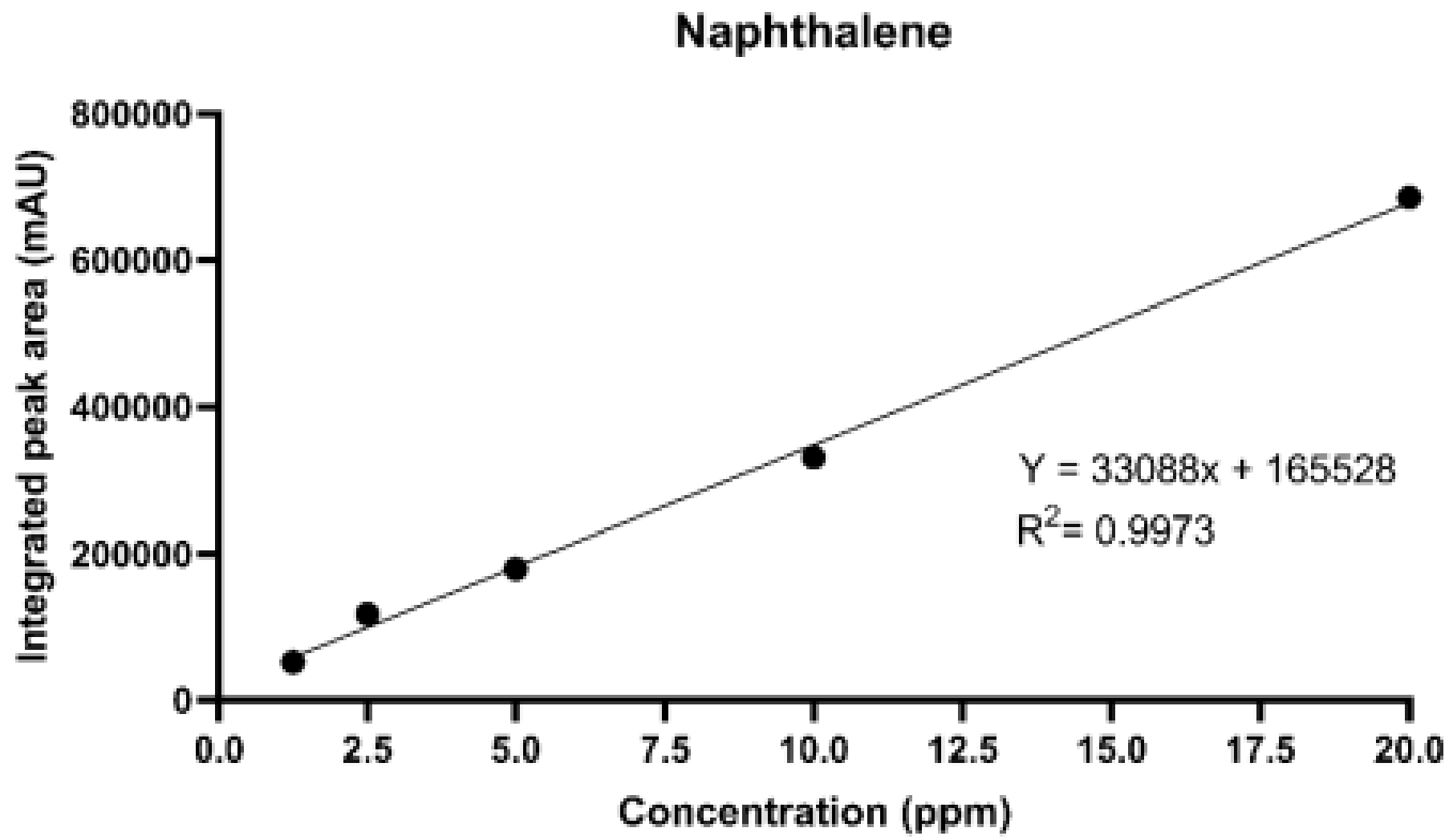

Figure S3. Calibration curve of naphthalene obtained using Ultra Fast Liquid Chromatography (UFLC). The calibration curve was constructed by plotting concentration (1.25-20 ppm) versus integrated peak area (mAU·s). Chromatographic separation was performed on a reversed-phase C18 column (150 mm × 4.6 mm, 5 µm) at 40°C using acetonitrile:water (70:30, v/v) under isocratic conditions with a flow rate of 1.0 mL/min. Detection was carried out at 270 nm. The regression equation was $y = 33088x + 165528$ with a correlation coefficient of $R^2 = 0.9973$, indicating good linearity.

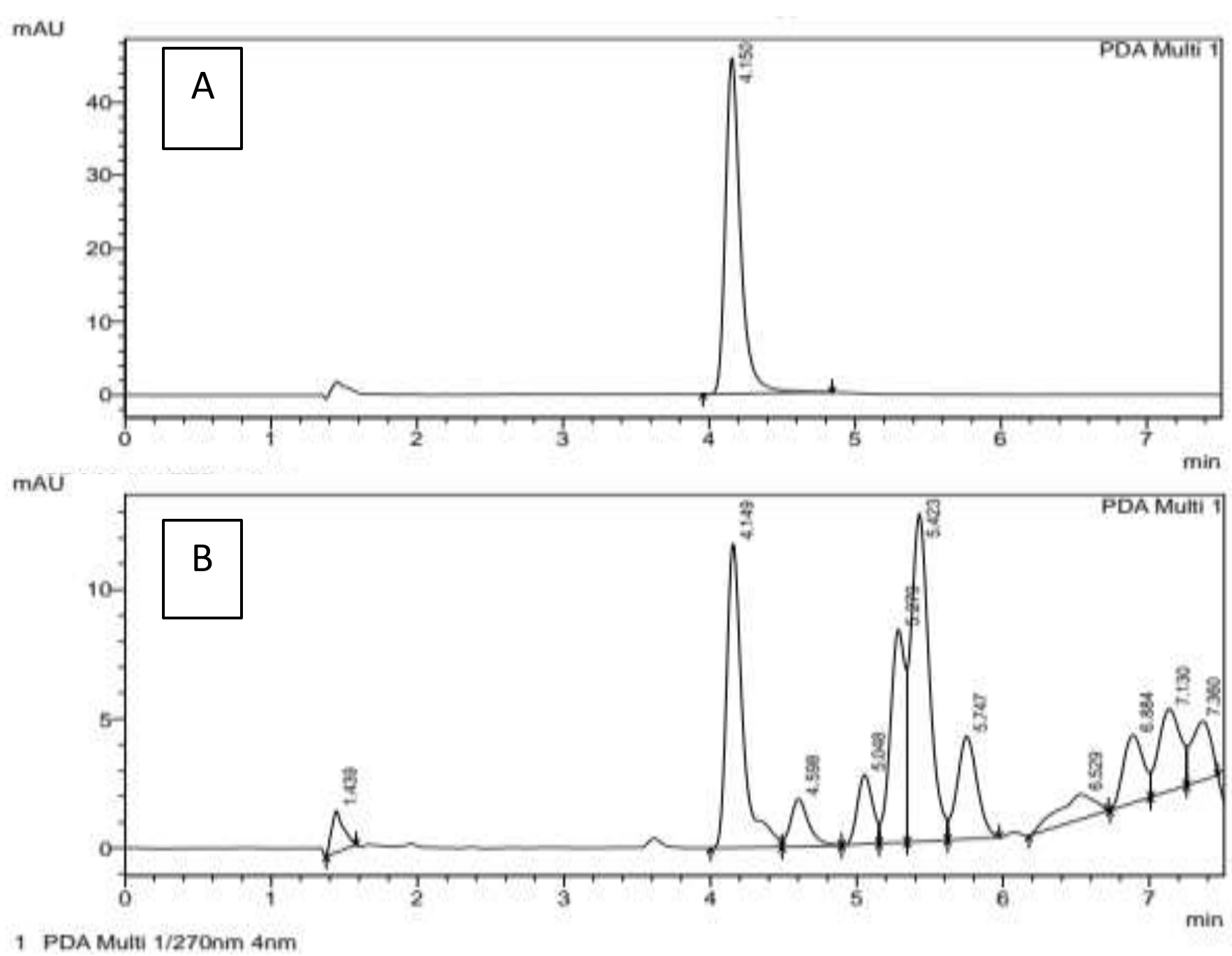

Figure S4. UFLC profiles of (A) naphthalene standard; and (B) Jet Fuel A-1 sample detected at 270 nm (PDA) showing peak of naphthalene at the retention time of 4.15 minutes.

Supporting Tables

Table S1. Calibration data of naphthalene standard solutions.

Concentration	Peak Area (mAU.s)	Retention Time (minute)
20	685329.6	4.147
10	330964.3	4.150
5	179134.2	4.151
2.5	117416.9	4.141
1.25	51973.7	4.145

Table S2. Quantification of naphthalene in Jet-A1 samples.

Sample	Peak Area (mAU.s)	Retention Time (minute)	Concentration (1.000 ppm)	Concentration (10.000 pm)	Jet-A1 0.5 mL concentration contains naphthalene
Jet-A1	85324	4.145	2.08	20.79	10.40
Jet-A1	89936.8	4.149	2.22	22.19	11.09
Jet-A1	93294.6	4.149	2.32	23.20	11.60
